# Efficacy comparison of radical antegrade modular pancreatosplenectomy versus conventional distal pancreatectomy in the treatment of left-sided pancreatic cancer: a meta-analysis and systematic review

**DOI:** 10.3389/fonc.2026.1782903

**Published:** 2026-06-12

**Authors:** Jincheng Wang, Guangqiang Gu, Hao Zhang, Furui Zhong

**Affiliations:** 1 Department of Hepatobiliary and Pancreatic Surgery, The Third Hospital of Mianyang, Sichuan Mental Health Center, Mianyang, Sichuan, China; 2 Department of Hepatobiliary and Pancreatic Surgery, Meishan City People’s Hospital, Meishan, Sichuan, China; 3 Department of General Surgery, Zigong Fourth People’s Hospital, Zigong, Sichuan, China

**Keywords:** distal pancreatectomy, left-sided pancreatic cancer, meta-analysis, radical antegrade modular pancreatosplenectomy, systematic review

## Abstract

**Objective:**

To systematically evaluate the efficacy and safety of radical antegrade modular pancreatosplenectomy (RAMPS) versus conventional distal pancreatectomy (CDP) for left-sided pancreatic cancer.

**Methods:**

Computerized searches were conducted in PubMed, Embase, Cochrane Library, CNKI, Wanfang Data and other Chinese and English databases to collect studies comparing RAMPS with CDP for left-sided pancreatic cancer published from January 2003 to October 2025. Two researchers independently completed literature screening and data extraction; the ROBINS-I tool was used for bias risk assessment; RevMan 5.4.1 software was used for effect size combination, and R software was used for sensitivity analysis and publication bias analysis.

**Result:**

A total of 21 studies were included, involving 2,591 patients, among which 9 were propensity score-matched (PSM) studies. The results of the meta-analysis showed that compared with CDP, RAMPS could significantly increase the R0 resection rate (RR = 1.10, 95% CI: 1.03-1.18, P = 0.007) and the number of lymph nodes dissected (MD = 3.58, 95%CI 1.66 – 5.50, P<0.001). However, substantial heterogeneity was noted for these outcomes (I2 = 72%/93%). No significant differences were found in operative time, blood loss, hospital stay, overall complication rates, or perioperative mortality. Crucially, there were no significant differences in overall survival (HR = 0.93, 95% CI: 0.76-1.13, P = 0.46) or disease-free survival (HR = 1.01, 95% CI: 0.77-1.33, P = 0.92) between the two procedures.

**Conclusion:**

For left-sided pancreatic cancer, RAMPS may offer advantages in local tumor control and surgical radicality; however, such advantages have not been shown to translate into meaningful improvements in survival outcomes.

## Introduction

Pancreatic cancer is a highly aggressive malignant tumor of the digestive system, with its global incidence exhibiting a continuous upward trend (1). In 2024, the number of new pancreatic cancer cases surpassed 500,000, while its overall 5-year survival rate is only approximately 9% (2), ranking it the seventh leading cause of cancer-related mortality (3). Among all pancreatic cancer cases, left-sided pancreatic cancer accounts for roughly 25%-30% (4). Due to the deep anatomical location of the pancreatic body and tail and the non-specificity of early symptoms (5), the majority of patients are diagnosed at the stage of local invasion or regional lymph node metastasis, with only 20%-30% being eligible for radical surgical resection (6). Currently, surgical resection remains the sole modality with the potential to achieve clinical cure, its core goal is to achieve R0 resection and complete regional lymph node dissection, so as to reducing the risk of local recurrence and improving the long-term prognosis of patients (7).

Conventional distal pancreatectomy (CDP) is the traditional standard surgical procedure for left-sided pancreatic cancer (8), which mainly involves the combined resection of the distal pancreatic body, tail, and spleen. In clinical practice, a “left-to-right” retrograde dissection approach is commonly adopted: the pancreatic body and tail are first gradually dissected from surrounding tissues including the posterior gastric wall and transverse mesocolon, followed by the management of splenic arteriovenous branches, and finally transection of the pancreatic neck to finalize the resection (9). However, this procedure has several clinical limitations: First, the resection plane is mostly confined to the posterior aspect of the pancreatic capsule, resulting in a high positive rate of retroperitoneal margins (10). Second, the lymph node dissection range usually only covers regions such as stations 10, 11, and 18, making it challenging to completely resect key draining lymph nodes around the celiac axis and superior mesenteric artery, this may increase the risk of underestimating tumor staging (11). Finally, its retrograde dissection path may cause intraoperative tumor compression, which could potentially increase the risk of hematogenous metastasis (12). These factors result in a local recurrence rate of 35%-50% after CDP (13), with a 5-year survival rate remaining at 15%-20% for a long time (14).

To overcome the radical bottleneck of traditional procedures, Strasberg et al. first proposed radical antegrade modular pancreatosplenectomy (RAMPS) in 2003 (15). This procedure is centered on the core design concepts of “antegrade dissection, modular lymph node dissection, and extended retroperitoneal resection”: the antegrade left-to-right resection approach avoids tumor compression; an anatomical plane is established posterior to Gerota’s fascia to expand the resection range of the retroperitoneal margin; and lymph nodes in the celiac axis, superior mesenteric artery, and Heidelberg triangle regions are dissected synchronously to achieve complete clearance of N1-N2 station lymph nodes (16). Theoretically, RAMPS can significantly improve the R0 resection rate, reduce the risk of positive margins, and refine tumor staging assessment through more comprehensive lymph node dissection, thus providing a precise basis for the formulation of postoperative adjuvant therapy regimens.

In recent years, multiple retrospective cohort studies have compared the clinical efficacy of RAMPS and CDP, but the conclusions remain highly controversial. Regarding surgical radicality, some studies have confirmed that RAMPS can increase the R0 resection rate to over 85% (17) and increase the number of dissected lymph nodes by 4-6 (18), while Takahashi et al. and Kwon et al. studies (19, 20) have found no statistically significant difference in R0 resection rates between the two groups. In terms of long-term survival, Li-pen et al. (21) found that the 5-year overall survival rate in the RAMPS group was 14.5% higher than that in the conventional procedure group, but a large-sample propensity score matching (PSM) study by Yin et al. (22) did not observe a significant difference in survival outcomes between the two groups. In terms of postoperative safety, owing to the expanded surgical scope of RAMPS, Li et al. studies (18) have reported slightly higher incidences of chylous leakage and delayed gastric emptying, but studies by Kim et al. and Dai et al. (10, 23) have confirmed that its total complication rate is comparable to that of the conventional procedure.

Currently, the evidence-based medicine level of relevant evidence is generally low, which is insufficient to form unified clinical guideline recommendations, resulting in significant differences in procedure selection among various centers. Therefore, it is necessary to integrate existing comparative study data through systematic review and meta-analysis to comprehensively and objectively evaluate the key outcome differences between RAMPS and CDP in the treatment of left-sided pancreatic cancer. This study adopts strict evidence-based medicine methods, aiming to synthesize current evidence, clarify the clinical advantages and limitations of RAMPS, and provide a high-quality reference for the optimization of surgical treatment strategies for left-sided pancreatic cancer.

## Methods

### Study design and registration

This study is a systematic review and meta-analysis, conducted strictly in accordance with the Preferred Reporting Items for Systematic Reviews and Meta-Analyses (PRISMA) statement (24). The study protocol has been registered on the International Prospective Register of Systematic Reviews (PROSPERO) (registration number: CRD420251269709), and all analytical procedures were consistent with the prespecified protocol without substantive modifications.

### Inclusion and exclusion criteria

#### Inclusion criteria

Participants (P): Patients with pathologically confirmed left-sided pancreatic cancer, without distant metastasis or severe organ dysfunction before surgery.

Intervention (I): The experimental group (RAMPS group) underwent radical antegrade modular pancreatosplenectomy, including laparoscopic RAMPS or open RAMPS, with surgical procedures conforming to the core criteria proposed by Strasberg: antegrade dissection, modular lymph node dissection, and resection plane posterior to Gerota’s fascia.

Comparator (C): The control group (CDP group) underwent conventional distal pancreatectomy, including laparoscopic/open distal pancreatectomy with splenectomy or standard retrograde pancreatosplenectomy (SRPS), without adopting the dissection and lymph node dissection principles of RAMPS.

Outcomes (O): At least one of the following core outcomes was reported: Surgical indicators: operation time, intraoperative blood loss, intraoperative transfusion rate, length of hospital stay; Oncological indicators: R0 resection rate, negative retroperitoneal margin rate, number of lymph nodes dissected; Postoperative complications: total complication rate, pancreatic fistula, bleeding, etc.; Survival indicators: perioperative mortality, overall survival (OS), disease-free survival (DFS). OS was defined as the time from surgery to death from any cause. DFS was defined as the time from the surgery to tumor recurrence, patient death, or the last follow-up.

Study design (S): Published retrospective cohort studies, prospective cohort studies, or randomized controlled trials (RCTs) with a direct comparative design evaluating the efficacy of RAMPS versus conventional distal pancreatectomy for left-sided pancreatic cancer.

Exclusion Criteria: (1) Review articles, case reports, or studies without direct comparison between RAMPS and CDP; (2) Studies that only descriptively reported the safety or efficacy of RAMPS or SRPS without providing specific comparable data; (3) Duplicate publications: only the study with the most complete data and reliable reporting was included to avoid bias caused by duplicate data entry.

### Literature search strategy

Comprehensive searches were performed in English databases (PubMed, Embase, Cochrane Library, Web of Science) and Chinese databases (China National Knowledge Infrastructure [CNKI], Wanfang Data Knowledge Service Platform, VIP Database). The search period was from January 2003 to October 2025. Search terms included: “pancreatic cancer”, “pancreatic body and tail cancer”, “distal pancreatic cancer”, “radical antegrade modular pancreatosplenectomy”, “RAMPS”, “distal pancreatectomy”, “conventional distal pancreatectomy”. Combined with MeSH terms (for PubMed) and free-text terms, using Boolean operators “AND/OR/NOT” to construct search strategies. **Supplementary Table 1** presents the database-specific search strategies. Hand searches of reference lists of included studies and relevant high-impact journals (published in the past 5 years) were conducted, and gray literature was also retrieved to avoid missing eligible studies.

### Literature screening and data extraction

Two independent researchers imported search results into EndNote X9 software, first removing duplicate literature, then performing initial screening based on titles and abstracts, and finally reviewing full texts to determine eligibility. Disagreements were resolved through arbitration by a third researcher. A pre-designed standardized data extraction form was used for independent data extraction by two researchers, followed by cross-verification.

Extracted data included: Basic study information: author, year of publication, country/region, study design, sample size; Baseline characteristics: age, gender, BMI, and tumor size of patients in both groups; Outcome indicators: surgical indicators, oncological indicators, postoperative complications and survival indicators. If hazard ratio (HR) and 95% CI were not reported for survival data, two researchers independently extracted data from Kaplan-Meier curves using Engauge Digitizer 4.1 software, following the method described by Wang et al. (25). Discrepancies >5% were resolved by consensus or arbitration by a third researcher.

### Study quality assessment

For included non-randomized controlled studies, the Risk of Bias in Non-randomized Studies of Interventions (ROBINS-I) tool was used to assess the risk of bias. The assessment covered seven core domains: confounding, selection of participants, classification of interventions, missing data, measurement of outcomes, reporting bias, and other biases. Two independent researchers completed the assessment and cross-verified the results; disagreements were resolved through discussion or arbitration by a third researcher. Finally, each study was classified into one of four risk levels: “low risk”, “moderate risk”, “high risk”, or “critical risk” based on the assessment results.

### Evaluation of the certainty of evidence

The Grading of Recommendations, Assessment, Development, and Evaluations (GRADE) framework (26) was used to assess the quality of evidence for the included outcomes. This approach considers factors such as risk of bias, inconsistency, imprecision, indirectness, and publication bias. Following the GRADE guidelines, two independent reviewers evaluated these domains and classified the certainty of evidence for each outcome as “high”, “moderate”, “low”, or “very low”.

### Statistical analysis

Statistical analyses were performed using RevMan 5.4 software and R software. For categorical variables (e.g., R0 resection rate, complication rate), the relative risk (RR) with 95% CI was used as the pooled effect size. For continuous variables (e.g., operation time, intraoperative blood loss, length of hospital stay), the mean difference (MD) with 95% CI was used. For survival outcomes, the HR with 95% CI was used. Heterogeneity was assessed using the Q test and I^2^ statistic: I^2^ <50% and P >0.10 indicated low heterogeneity, while the opposite indicated high heterogeneity. A random-effects model was used for meta-analysis to fully account for clinical and methodological heterogeneity between studies. Sensitivity analysis was performed by sequentially excluding each individual study and re-pooling effect sizes to verify the stability of the results. For outcomes with ≥10 included studies, funnel plots were used to visualize publication bias, and Egger’s test was used to quantify the degree of bias.

## Results

### Literature search

**Figure 1:**
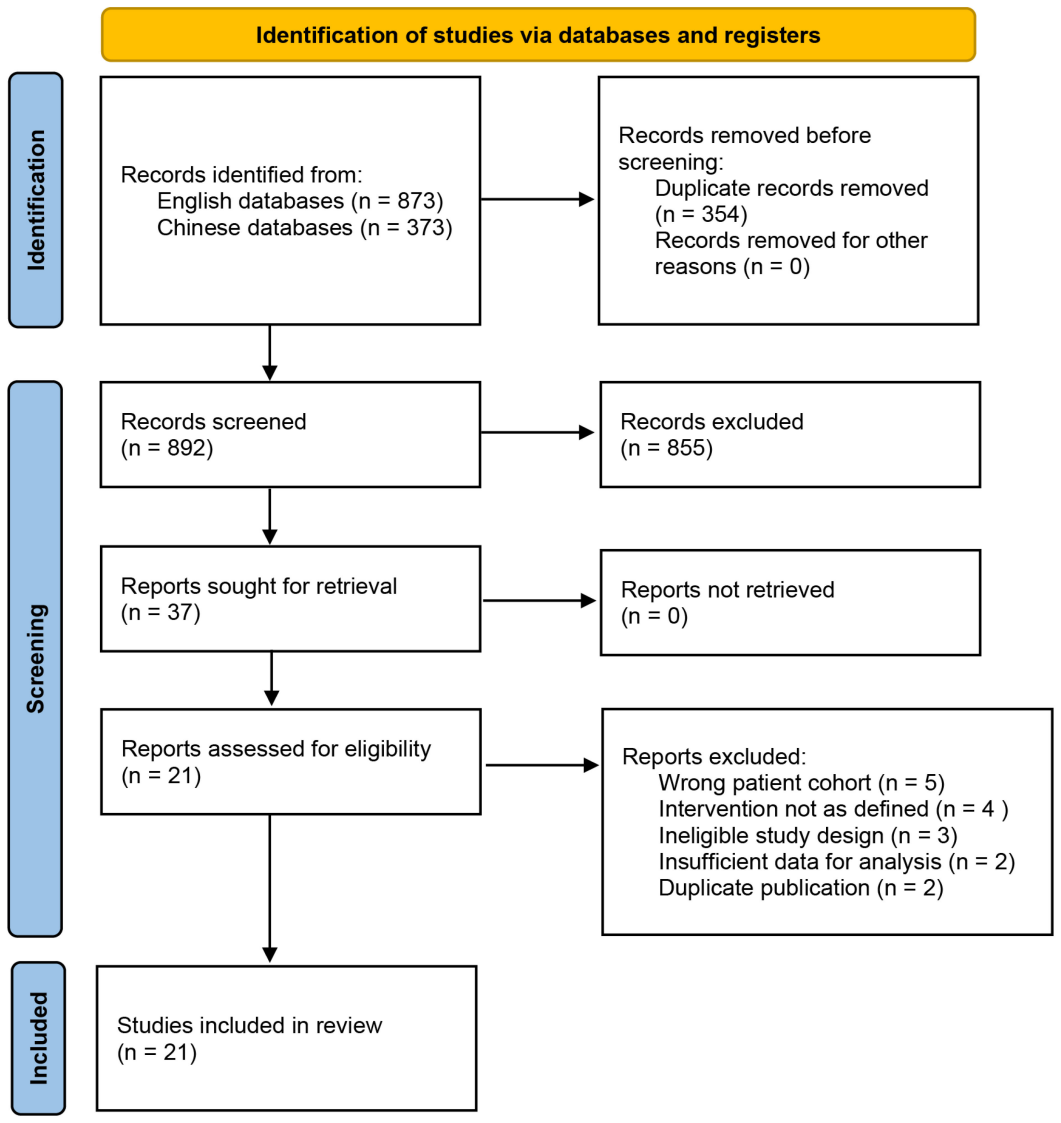
Flow diagram of studies included in meta-analysis.

Following the prespecified search strategy, an initial search yielded 1246 relevant articles (873 in English, 373 in Chinese). After deduplication using EndNote X9, 892 articles remained. Through initial screening based on titles and abstracts, 765 articles were excluded, including non-comparative studies and those with incomplete data. After further full-text review for secondary screening, 21 eligible clinical studies (10, 17–23, 27–39) were finally included, all of which were retrospective comparative studies. The search process is shown in **Figure 1**.

### Baseline characteristics of included studies

The 21 included studies were published between 2013 and 2025, with 10 studies (10, 18, 21, 22, 27–30, 35, 39) conducted in China, 5 studies (20, 23, 33, 36, 37) in South Korea, 2 studies (19, 31) in Japan, 1 study (32) in the United States, 1 study (38) in Italy, 1 study (34) in Poland, and 1 study (17) including participants from both China and the United States. Among them, 8 studies (18–20, 22, 23, 29, 32, 36) used data after PSM, and 13 studies used overall sample data. The total sample size was 2591 cases, including 1282 cases in the RAMPS group and 1309 cases in the control group; the sample size of each study ranged from 25 to 446 cases. The baseline characteristics of the included studies are shown in **Table 1**.

**Table 1:** Baseline characteristics of the 21 included studies.

First author	Year	Country	Study type	Sample size		Age (years)		Sex (M/F, n)		Tumor diameter (cm)		Follow-up period (month)	
				RAMPS	CDP	RAMPS	CDP	RAMPS	CDP	RAMPS	CDP	RAMPS	CDP
Latorre et al. (38)	2013	Italy	Retrospective	8	17	61	60	5/3	11/6	4.9	5.2	NA	NA
Lee et al. (36)	2014	South Korea	PSM	10	40	63.3 ± 9.9	62.7 ± 9.1	6/4	25/15	2.3 ± 0.6	3.2 ± 1.5	>36	
Park et al., (37)	2014	South Korea	Retrospective	38	54	62.2± 8.7	61.3 ± 10.5	23/15	35/19	3.1	3.8	18(3-82)	16(4-148)
Abe et al. (31)	2016	Japan	Retrospective	53	40	68.6 ± 10.7	65.2 ± 8.6	34/22	29/11	NA	NA	20.5	28.6
Kim et al. (33)	2016	South Korea	Retrospective	30	19	63.7 ± 8.2	62.1 ± 8.5	13/17	7/12	4.6 ± 1.6	4.5 ± 1.5	33 (8-97)	
Xu et al. (27)	2016	China	Retrospective	21	78	62 ± 11	63 ± 9	11/10	41/37	5.0 ± 1.7	3.8 ± 1.5	18 (5-37)	
Wang et al. (39)	2018	China	Retrospective	30	30	48-76		34/26		6.2 ± 3.2		NA	NA
Huo et al. (30)	2019	China	Retrospective	11	16	63.9 ± 3.2	63.3 ± 2.6	8/3	5/11	3.7 ± 0.2	4.4 ± 0.6	NA	NA
Yin et al. (29)	2020	China	PSM	81	81	65.6 ± 8.8	63.7 ± 9.1	44/37	50/31	NA	NA	16.3 (3.0-49.6)	
Sham et al. (17)	2020	China + USA	Retrospective	253	193	55 ± 20.7	69 ± 9.3	151/102	100/93	4.8 ± 1.7	3.9 ± 1.6	24.3	
Dai et al. (10)	2021	China	Retrospective	46	57	62.0 ± 8.8	62.2 ± 10.2	23/23	28/29	4.3 ± 2.0	4.2 ± 1.8	78.6	
Kim et al. (23)	2021	South Korea	PSM	37	37	67.3 ± 9.3	64.3 ± 10.8	13/24	21/16	3.8 ± 1.4	3.9 ± 2.3	NA	NA
Niu et al. (28)	2022	China	Retrospective	50	59	67 ± 10.7	66 ± 12.5	31/19	37/22	3.5 ± 2.1	3.5 ± 1.4	46.7	
Sutton et al. (32)	2022	USA	PSM	89	89	60 ± 6	60 ± 5.5	42/47	39/50	NA	NA	17 (1-101)	
Takahashi et al. (19)	2023	Japan	PSM	54	54	69 ± 13.3	67 ± 10.1	36/18	39/15	3.3 ± 3.2	3.5 ± 1.4	32 (3-134)	
Zhu et al. (35)	2023	China	Retrospective	44	39	64.6 ± 8.8	63.2 ± 8.6	27/17	24/15	3.4 ± 1.4	4.1 ± 2.2	NA	NA
Borys et al. (34)	2024	Poland	Retrospective	12	13	70 ± 5.9	65 ± 7.4	2/10	6/7	2.6 ± 1.7	4.5 ± 1.1	23 (15-31)	59 (43-71)
Kwon et al. (20)	2024	South Korea	PSM	99	99	66.0 ± 9.5	65.0 ± 10.3	53/46	53/46	3.2 ± 1.8	3.0 ± 1.8	20 (3-133)	26 (3-133)
Li et al. (18)	2024	China	PSM	54	54	67.0 ± 3.3	67.2 ± 3.2	31/23	28/26	3.0 ± 1.0	3.0 ± 1.0	20.4	
Li-pen et al. (21)	2024	China	Retrospective	88	66	64.38 ± 8.21	62.64 ± 8.52	34/54	27/39	2.9 ± 1.0	2.6 ± 1	25	13
Yin et al. (22)	2025	China	PSM	174	174	65.3 ± 9.5	64.0 ± 9.5	101/73	102/72	NA	NA	25.6 (16.8-43.4)	

PSM, Propensity score matching; RAMPS, Radical antegrade modular pancreatosplenectomy; CDP, Conventional distal pancreatectomy; NA, Not available; M/F, Male/Female.

### Risk of bias

The ROBINS-I tool was used to assess the risk of bias for the 21 included non-randomized controlled studies. Regarding confounding bias, the 9 studies using PSM balanced key covariates such as age and tumor stage, and were all rated as low risk of bias. For selection bias, all studies were based on case selection from single or multiple centers without a clear random sampling process, resulting in a certain degree of selection bias and thus rated as moderate risk. Classification bias of interventions was low risk, as the definitions of RAMPS and conventional distal pancreatectomy were clear and the classification criteria were consistent. The risks of missing data bias, measurement bias, and reporting bias were mostly low. Overall, the risk of bias for all included studies was moderate (**Supplementary Table 2**).

### Surgical-related outcomes

**Figure 2:**
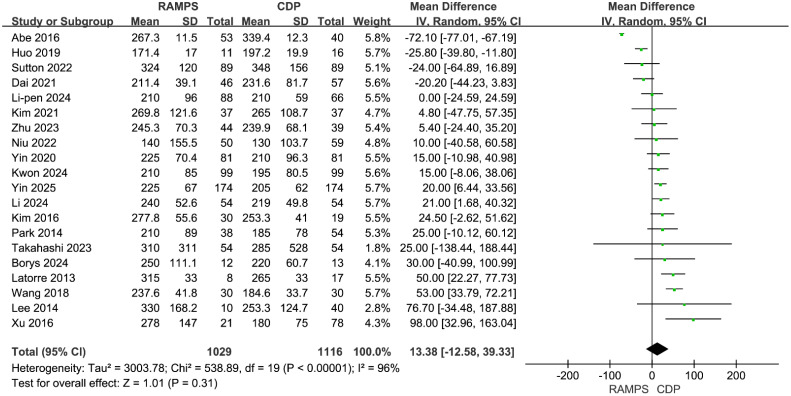
Forest plot of comparison of RAMPS versus CDP for operative time.

**Figure 3:**
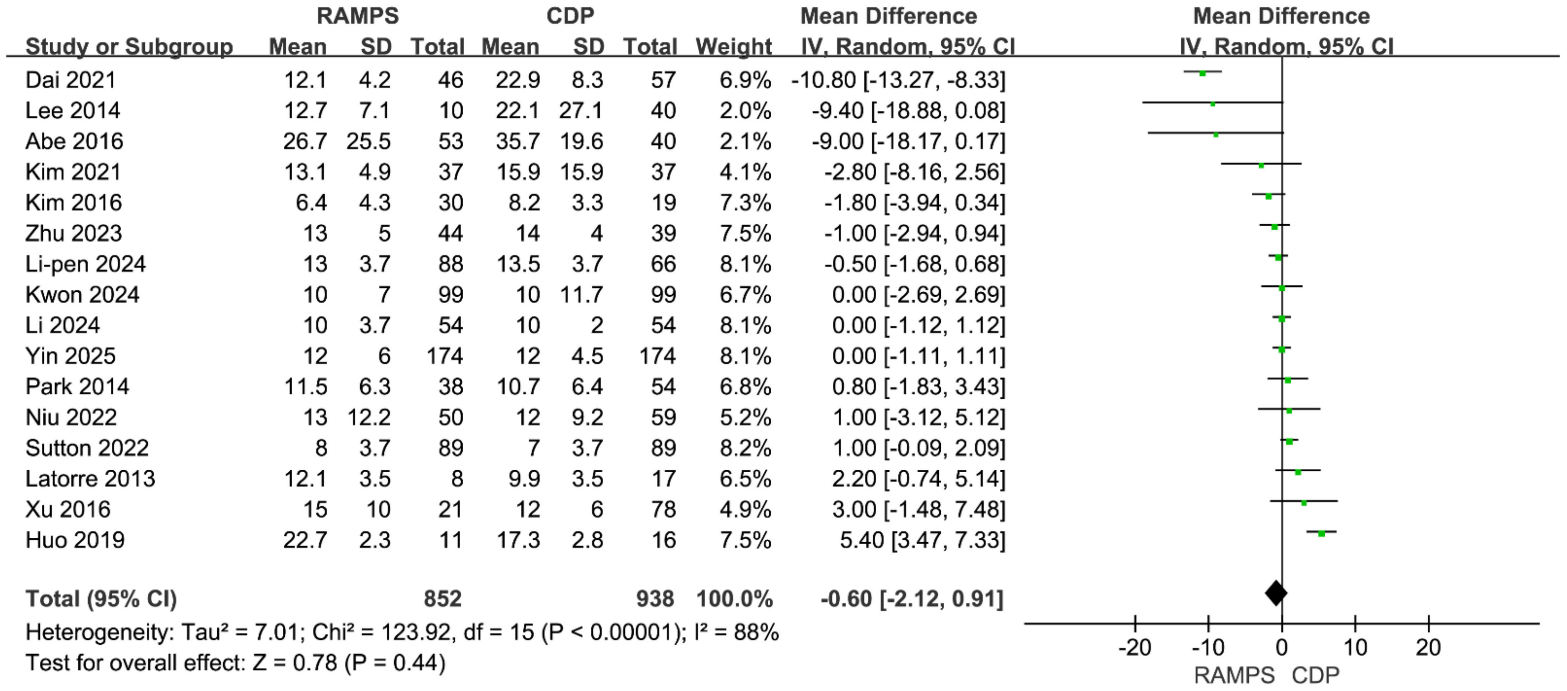
Forest plot of comparison of RAMPS versus CDP for length of hospital stay (Effect size: MD; MD < 0 favors RAMPS).

Among the included studies, 20 studies (10, 18–23, 27–39) reported operation time, 20 studies (10, 17–23, 27–37, 39) reported intraoperative blood loss, 10 studies (10, 18, 20, 21, 23, 29, 31, 35, 36, 38) reported intraoperative transfusion rate, and 16 studies (10, 18, 20–23, 27, 28, 30–33, 35–38) reported the length of hospital stay. Heterogeneity tests showed high heterogeneity for operation time (I^2^ = 96%), intraoperative blood loss (I^2^ = 94%), and length of hospital stay (I^2^ = 88%), while intraoperative transfusion rate had low heterogeneity (I^2^ = 0%). Meta-analysis results indicated no significant differences between the RAMPS group and CDP group in operation time (MD = 13.38, 95%CI -12.58 – 39.33, P = 0.31) (**Figure 2**), intraoperative blood loss (MD=-43.37, 95%CI -97.83 – 11.09, P = 0.12) (**Supplementary Figure 1**), intraoperative transfusion rate (RR = 0.79, 95%CI 0.58 – 1.07, P = 0.13) (**Supplementary Figure 2**), or length of hospital stay (MD=-0.60, 95%CI -2.12 – 0.91, P = 0.44) (**Figure 3**).

### Oncological outcomes

**Figure 4:**
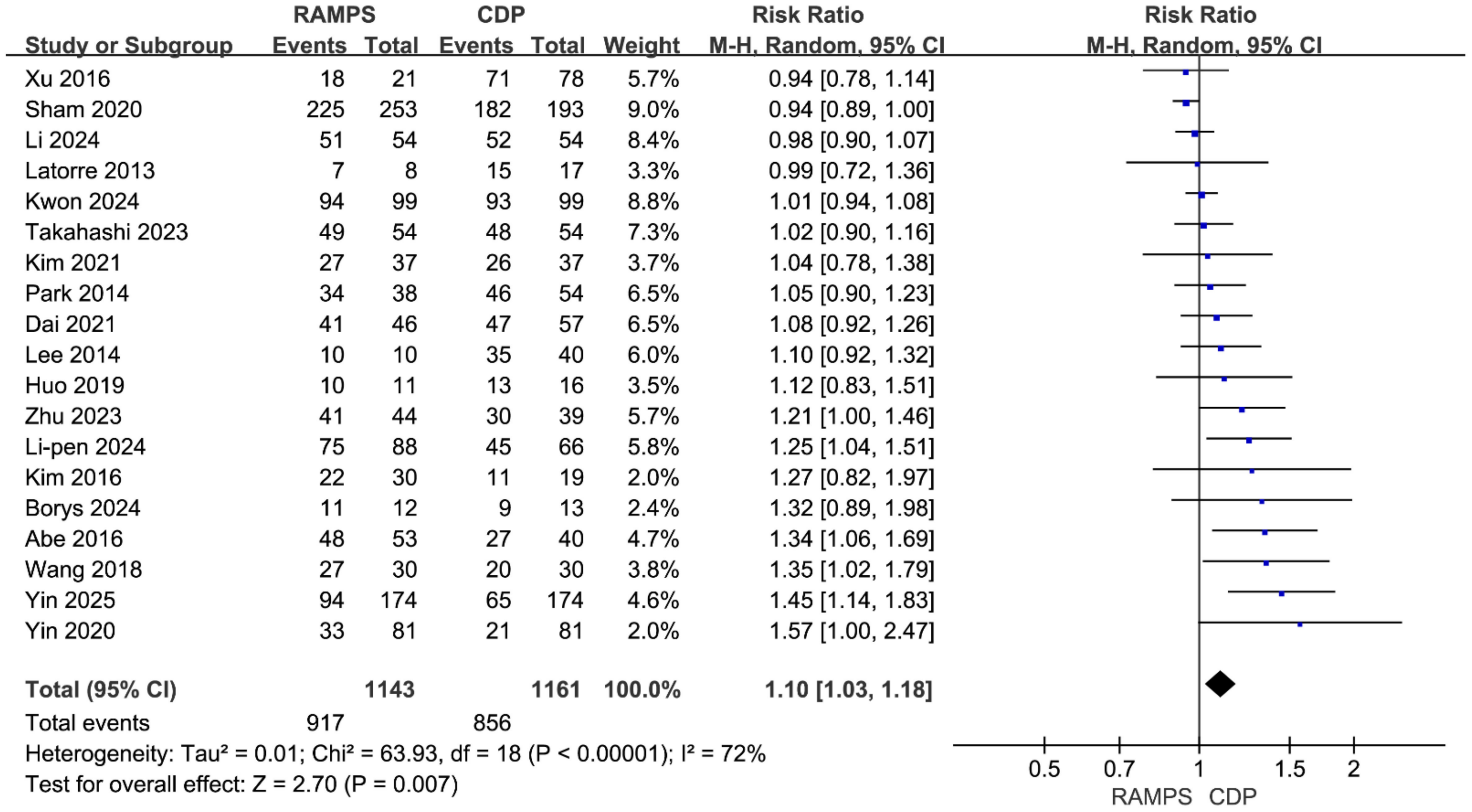
Forest plot of comparison of RAMPS versus CDP for R0 resection rate.

**Figure 5:**
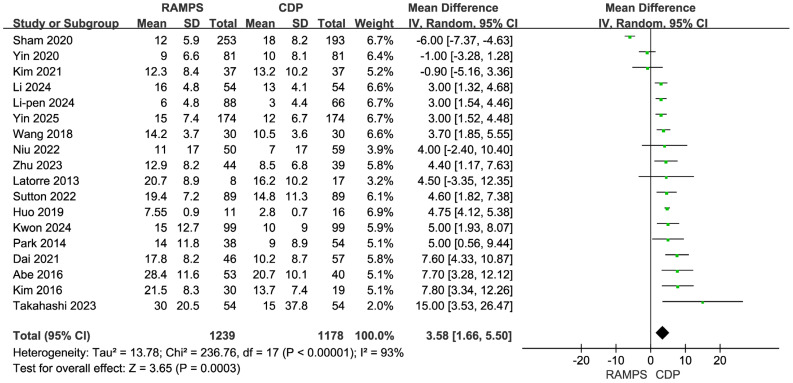
Forest plot of comparison of RAMPS versus CDP for number of lymph nodes dissected.

Nineteen studies (10, 17–23, 27, 29–31, 33–39) reported R0 resection rate, 18 studies (10, 17–23, 28–33, 35, 37–39) reported the number of lymph nodes dissected, and 8 studies (10, 18, 21, 23, 29, 30, 36, 38) reported negative retroperitoneal margin rate. Heterogeneity tests showed high heterogeneity for all three outcomes: R0 resection rate (I^2^ = 72%), number of lymph nodes dissected (I^2^ = 93%), and negative retroperitoneal margin rate (I^2^ = 72%). Meta-analysis results demonstrated that the RAMPS group was significantly superior to the CDP group in R0 resection rate (RR = 1.10, 95%CI 1.03 – 1.18, P = 0.007) (**Figure 4**), number of lymph nodes dissected (MD = 3.58, 95%CI 1.66 – 5.50, P<0.001) (**Figure 5**), and negative retroperitoneal margin rate (RR = 1.20, 95%CI 1.06 – 1.36, P = 0.005) (**Supplementary Figure 3**).

### Postoperative complications

Fifteen studies (10, 17, 18, 21–23, 27, 29, 31, 34–39) reported total complications, 14 studies (18–21, 23, 27, 28, 31, 33–38) reported Clavien-Dindo ≥ grade III complications, 17 studies (10, 17, 19–21, 23, 27, 28, 30, 31, 33–39) reported postoperative pancreatic fistula, 15 studies (10, 17, 18, 20, 21, 27–29, 31, 33–39) reported postoperative bleeding, 12 studies (10, 20, 22, 27, 29, 31, 33–38) reported delayed gastric emptying, and 9 studies reported (18, 20, 27, 31, 33, 36–38) chylous leakage. Heterogeneity tests showed high heterogeneity for total complications (I^2^ = 79%), while the remaining postoperative complication outcomes had low heterogeneity. Meta-analysis results showed no significant differences in postoperative complications between the RAMPS group and CDP group (**Table 2**).

**Table 2:** Meta-analysis results of postoperative complications.

Postoperative complications	Study (n)	Heterogeneity (I^2^)	Effect size (RR)	95% CI	P
Postoperative pancreatic fistula	17	27%	1.01	0.75 - 1.36	0.93
Postoperative bleeding	15	0%	0.86	0.51 - 1.42	0.55
Delayed gastric emptying	12	9%	1.15	0.64 - 2.08	0.63
Chylous leakage	9	0%	1.36	0.76 - 2.44	0.29
Clavien-Dindo ≥ grade III	14	0%	1.19	0.78 - 1.82	0.42
Total complications	15	79%	0.83	0.51 - 1.34	0.44

### Survival outcomes

**Figure 6:**
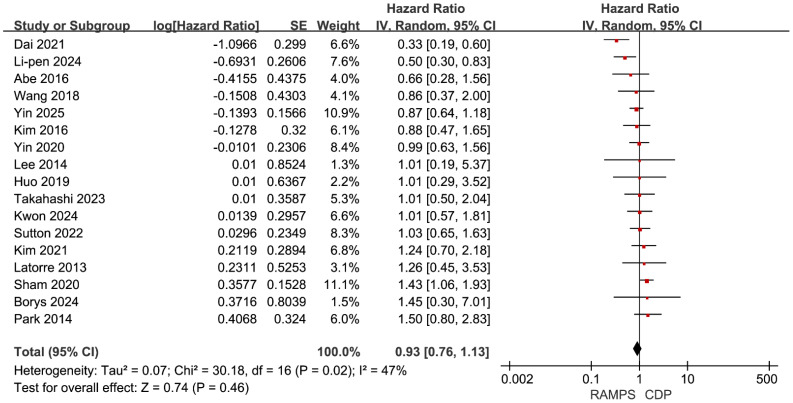
Forest plot of comparison of RAMPS versus CDP for overall survival.

**Figure 7:**
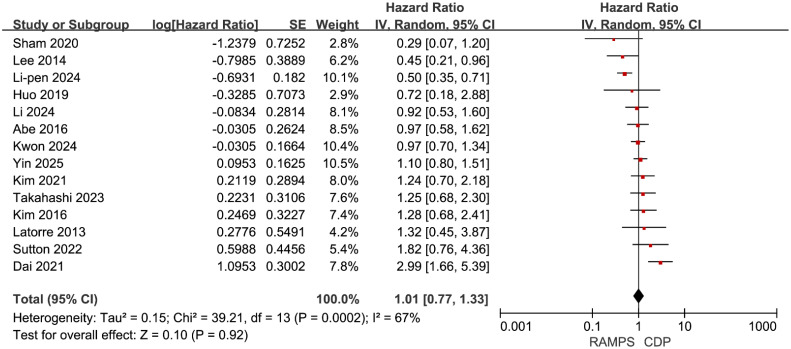
Forest plot of comparison of RAMPS versus CDP for disease-free survival.

Twenty studies (10, 17–23, 27–29, 31–39) reported perioperative mortality, 17 studies (10, 17, 19–23, 29–34, 36–39) reported OS, and 14 studies (10, 17–23, 30–33, 36, 38) reported DFS. Heterogeneity tests showed high heterogeneity for DFS (I^2^ = 67%), while perioperative mortality (I^2^ = 0%) and OS (I^2^ = 46%) had low heterogeneity. Meta - analysis findings demonstrated no significant differences in perioperative mortality (RR = 1.16, 95%CI 0.30 – 4.52, P = 0.83) (**Supplementary Figure 4**), OS (HR = 0.93, 95%CI 0.76 – 1.13, P = 0.46) (**Figure 6**) and DFS (HR = 1.01, 95%CI 0.77 – 1.33, P = 0.92) (**Figure 7**) between the RAMPS group and the CDP group.

### Sensitivity analysis

Sensitivity analysis was performed by sequentially excluding each individual study to verify the stability of the results. After excluding the study by Abe et al. (31), the point estimate and 95% CI of the pooled effect size for operation time changed significantly (MD = 16.00; 95%CI 2.39 – 29.61; P = 0.02). After excluding the study by Kim et al. (33), the point estimate and 95% CI of the pooled effect size for intraoperative transfusion rate changed significantly (RR = 0.70; 95% CI 0.50 - 0.99; P = 0.04). For the remaining outcomes, no significant changes in the point estimate or 95% CI of the pooled effect size were observed after excluding any single study.

### Publication bias

Formal Egger’s regression tests and funnel plots were performed only for outcomes with ≥10 included studies, as funnel plots become unreliable with fewer studies. These outcomes included R0 resection rate (19 studies), number of lymph nodes dissected (18 studies), overall survival (17 studies), postoperative pancreatic fistula (17 studies), postoperative bleeding (15 studies), disease-free survival (14 studies), total complications (15 studies), and perioperative mortality (20 studies). Visual inspection of the funnel plots did not reveal any obvious asymmetry (**Supplementary Figure 5**). None of the Egger’s regression tests reached statistical significance (P>0.05), suggesting no strong evidence of small-study effects.

### Evidence quality assessment

The GRADE approach was used to assess the certainty of evidence for all outcomes in this study. Given that all included studies were observational and substantial heterogeneity was present across most outcomes, the certainty of evidence was downgraded for the majority of endpoints. Perioperative mortality remained at moderate certainty, as it is an objective rare event with negligible heterogeneity (I²=0%). The detailed GRADE ratings and downgrading justifications for each outcome are presented in **Supplementary Table 3**.

## Discussion

This meta-analysis systematically retrieved and synthesized data from 21 retrospective controlled studies involving 2,591 patients with left-sided pancreatic cancer, with the aiming to comprehensively compare the clinical efficacy of RAMPS and CDP. The findings revealed that RAMPS demonstrated improved surrogate measures of surgical radicality—including higher R0 resection rates(RR = 1.10, 95%CI 1.03 – 1.18, P = 0.007), increased the number of lymph nodes dissected (MD = 3.58, 95%CI 1.66 – 5.50, P<0.001), and improved negative retroperitoneal margin rates (RR = 1.20, 95%CI 1.06 – 1.36, P = 0.005)—compared with CDP. However, no statistically significant differences were observed between the two groups in surgery related parameters (e.g., operation time, intraoperative blood loss, length of hospital stay, intraoperative transfusion rate), postoperative complication rates, or survival endpoints (OS and DFS).

As a modified version of conventional distal pancreatectomy, RAMPS is designed to improve local tumor control by optimizing the surgical approach and extending the resection extent (19). The superiority in local tumor control confirmed in this meta - analysis is directly attributed to its technical features. RAMPS adopts an antegrade dissection strategy, progressing from the pancreatic neck to the left, and extends the posterior peritoneal resection plane to the level of Gerota’s fascia (36). This approach allows for better visualization of the retroperitoneal space and effective clearance of potential residual tumor tissues and invaded soft tissues, which is consistent with the single-center findings of Li et al (21). They reported that the R0 resection rate in the RAMPS group (85.23%) was significantly higher than that in the SPRS group (68.18%), and the negative posterior peritoneal margin rate reached 96.59% (vs. 75.76% in the SPRS group). For patients at high risk of local invasion, this advantage can directly reduce the risk of local recurrence and create more favorable conditions for subsequent adjuvant therapy (17). RAMPS routinely involves lymph node dissection in regions 7, 8, 9, and 14a/b, which is more extensive than the standard dissection scope of CDP (mainly focusing on groups 10, 11, and 18 lymph nodes) (40). This meta - analysis showed that the number of lymph nodes harvested in the RAMPS group was significantly higher, which not only improves the accuracy of tumor N-staging and avoids “false-negative” staging due to insufficient lymph node dissection but also reduces the risk of regional lymph node residue (41). Masuda et al.’s study (42) has confirmed that adequate lymph node dissection is crucial for accurate staging and prognosis evaluation after pancreatic cancer surgery, and the design of RAMPS precisely meets this clinical requirement.

Although RAMPS has a wider surgical scope and more complex anatomy, this meta-analysis demonstrated no significant differences between RAMPS and CDP in operative duration, intraoperative blood loss, hospital stay, or postoperative complication rates, confirming the clinical safety of this procedure. Since RAMPS routinely transects the pancreatic neck first and uses a right to left antegrade dissection sequence, it enables early proactive control of the major vessels of the spleen, kidney, and adrenal gland, which helps reduce intraoperative bleeding and shorten the operative duration (31). While some single-center studies (20, 21) have reported that RAMPS may increase the risk of chylous leakage due to extended lymph node dissection involving the pericaeliac lymphatic tissue, this meta-analysis found no statistically significant differences in the incidence of major complications such as pancreatic fistula, bleeding, and delayed gastric emptying. This may be due to the precise vascular management and standardized postoperative care (e.g., drainage tube placement, nutritional support, anti-infection treatment) in RAMPS (43), which effectively controls the occurrence and progression of complications.

In the contemporary treatment landscape of pancreatic cancer, the role of surgical resection has become increasingly integrated with multimodal therapy, including neoadjuvant chemotherapy and precision oncology strategies (44). Despite the clear advantages of RAMPS in local tumor control, this study did not observe significant improvements in OS and DFS, which is consistent with the conclusions of multi-center studies by Kwon et al. (20) and Yin et al (29). The core reasons for this inconsistency may include the following. First, the extended lymph node dissection in RAMPS may be considered “overtreatment”. According to the guidelines of the International Study Group of Pancreatic Surgery (ISGPS), the standard lymph node dissection scope for left-sided pancreatic cancer should focus on groups 10, 11, and 18 lymph nodes (41, 45). However, the lymph nodes in groups 7, 8, 9, and 14a/b, which are routinely dissected in RAMPS, are not mandatory for pancreatic body and tail cancer (42). The additional lymph node dissection does not provide survival benefits but may increase surgical trauma (46). A metaregression analysis by Ricci et al. (47) also confirmed that the survival advantage of RAMPS only appeared in studies with an imbalance in the proportion of T1 - T2 stage tumors, which was essentially likely to be a spurious association caused by selection bias. Second, the biological characteristics of pancreatic cancer determine the core of survival prognosis (48). For patients with lymph node metastasis or vascular invasion, their survival prognosis depends more on the intrinsic malignancy of the tumor and the efficacy of systemic therapy, and the local control advantage of RAMPS cannot be fully translated into survival benefits (20). Third, unlike pancreatic head cancer, a positive posterior peritoneal margin has a relatively limited impact on survival in pancreatic body and tail cancer (49). A population - based study by Aaquist et al. (50) confirmed that the resection margin status is not an independent prognostic factor for patients with pancreatic body and tail cancer, which explains why RAMPS, despite significantly increasing the negative posterior peritoneal margin rate, did not improve long - term survival. In addition, some studies have shown that slower gastrointestinal function recovery after RAMPS (e.g., increased daily bowel movements) may lead to delayed initiation or reduced completion rate of adjuvant chemotherapy, which to some extent weakens the potential survival advantage of the surgery (51). Kwon et al.’s study (20) has confirmed that adjuvant therapy is an independent prognostic factor for survival after left - sided pancreatic cancer surgery, and its impact may be more significant than the difference in surgical procedures (52). With the widespread adoption of neoadjuvant therapy and advances in precision oncology, systemic control has become a dominant determinant of long-term survival, often outweighing the impact of local surgical radicality (53).

Given the above findings, the selection between RAMPS and CDP should be individualized. The oncological benefits of RAMPS in achieving higher R0 and negative posterior peritoneal margin rates suggest that it may be most appropriate in specific clinical scenarios where optimal local control is paramount. Specifically, RAMPS should be preferentially considered in patients with radiologic suspicion of posterior pancreatic invasion, retroperitoneal soft tissue involvement, or a high risk of local recurrence based on preoperative imaging findings. For patients with larger tumors, higher T stage, or suspected lymph node metastasis around the celiac axis or superior mesenteric artery, the extended dissection plane and more comprehensive lymphadenectomy of RAMPS may provide superior oncological radicality compared with conventional CDP. In contrast, for small, early-stage (T1-T2) left-sided pancreatic cancers without evidence of posterior extension or nodal involvement, CDP may remain a sufficient and reliable surgical option. These clinical scenario-based recommendations may help surgeons individualize the surgical strategy and maximize the potential oncological benefits of RAMPS.

Several limitations of this study warrant careful consideration. Given that all included studies were retrospective in design, inherent selection and confounding biases could not be fully eliminated. Although a random-effects model was employed, the extremely high heterogeneity observed in metrics such as operative time (I²=96%), blood loss (I²=94%), and lymph node yield (I²=93%) limits the precision of the pooled estimates. Furthermore, due to difficulties in obtaining detailed data from the included studies, we were unable to conduct subgroup analyses or meta-regression based on tumor stage, surgical approach subtypes (open vs. laparoscopic), or adjuvant therapy regimens, which further limits the depth of our findings. Additionally, the pooling of survival data did not adequately account for variations in follow-up duration (range: 16.3–78.6 months) and heterogeneity in systemic therapies, while the lack of individual patient data precluded adjustment for key confounders such as CA19–9 levels and vascular invasion, necessitating cautious interpretation of survival benefits. Finally, sparse data for certain key outcomes (e.g., negative posterior peritoneal margin rate) and the pooling of open, laparoscopic, and robotic approaches further limit the certainty of our conclusions. Future high-quality randomized controlled trials and stratified analyses based on uniform surgical approaches are needed to validate these findings.

## Conclusion

The results of this meta-analysis suggest that RAMPS may offer advantages in local tumor control and surgical radicality; however, such advantages have not been shown to translate into meaningful improvements in long-term survival outcomes. This suggests that in future clinical practice, the choice of surgical approach should be based on a comprehensive consideration of the patient’s individual characteristics, tumor specific conditions, and the technical proficiency of the medical team. Meanwhile, the long - term efficacy of RAMPS and its impact on patients’ quality of life still need to be further verified and explored through more high-quality prospective RCTs.
