# Supplementary material for: Efficacy comparison of radical antegrade modular pancreatosplenectomy versus conventional distal pancreatectomy in the treatment of left-sided pancreatic cancer: a meta-analysis and systematic review
Source: Front Oncol. 2026 Jun 12;16:1782903. doi: 10.3389/fonc.2026.1782903 (PMC13303372; doi:10.3389/fonc.2026.1782903)

**Efficacy Comparison of Radical Antegrade Modular Pancreatosplenectomy versus Conventional Distal Pancreatectomy in the Treatment of Left-Sided Pancreatic Cancer: A Meta-Analysis and Systematic Review**

Supplemental Table S1. Search Strategy (Updated Search on 30st October 2025)

Database: PubMed

| Search no. | Indexed and keywords terms |
| --- | --- |
| #1 | (("Pancreatic Neoplasms"[MeSH Terms] OR "pancreatic cancer"[Title/Abstract] OR "pancreatic ductal adenocarcinoma"[Title/Abstract] OR "left-sided pancreatic cancer"[Title/Abstract] OR "pancreatic body and tail cancer"[Title/Abstract] OR "pancreatic carcinoma of body and tail"[Title/Abstract]) |
| #2 | ("Radical Antegrade Modular Pancreatosplenectomy"[Title/Abstract] OR "RAMPS"[Title/Abstract] OR "conventional distal pancreatectomy"[Title/Abstract] OR "distal pancreatectomy"[Title/Abstract] OR "distal pancreatosplenectomy"[Title/Abstract] OR "CDP"[Title/Abstract] OR "DPS"[Title/Abstract]) |
| #3 | (("controlled study"[pt] OR "randomized controlled trial"[pt] OR "retrospective study"[pt] OR "prospective study"[pt] OR "cohort study"[pt] OR "comparative study"[Title/Abstract] OR "case-control study"[pt]) NOT ("review"[pt] OR "meta-analysis"[pt] OR "systematic review"[pt] OR "comment"[pt] OR "letter"[pt])) AND  ("Humans"[MeSH Terms] AND "Adult"[MeSH Terms] OR "Aged"[MeSH Terms]) |
| #4 | #1 AND #2 AND #3 |

Database: Embase

| Search no. | Indexed and keywords terms |
| --- | --- |
| #1 | (('pancreatic neoplasms'/exp OR 'pancreatic cancer' OR 'pancreatic carcinoma' OR 'pancreatic body and tail cancer' OR 'left-sided pancreatic cancer' OR 'pancreatic adenocarcinoma') |
| #2 | ('radical antegrade modular pancreatosplenectomy' OR 'RAMPS' OR 'conventional distal pancreatectomy' OR 'distal pancreatectomy' OR 'distal pancreatosplenectomy' OR 'CDP' OR 'DPS') |
| #3 | (('comparative study' OR 'cohort study' OR 'randomized controlled trial' OR 'retrospective study' OR 'prospective study') NOT ('review' OR 'editorial' OR 'letter' OR 'case report' OR 'meta-analysis')) |
| #4 | ('humans'/exp) AND  ('adult'/exp OR 'middle aged'/exp OR 'aged'/exp)) |
| #5 | #1 AND #2 AND #3 AND #4 |

Database: Web of Science

| Search no. | Indexed and keywords terms |
| --- | --- |
| #1 | TS=((((pancreatic neoplasms OR pancreatic cancer OR pancreatic carcinoma OR pancreatic body and tail cancer OR left-sided pancreatic cancer OR pancreatic adenocarcinoma) AND (radical antegrade modular pancreatosplenectomy OR RAMPS OR conventional distal pancreatectomy OR distal pancreatectomy OR distal pancreatosplenectomy OR CDP OR DPS)) AND ((comparative study OR cohort study OR randomized controlled trial OR retrospective study OR prospective study) NOT (review OR meta-analysis OR systematic review OR editorial OR letter OR case report))) AND (humans) AND (adult OR middle aged OR aged)) |

Database: cochrane library (CENTRAL)

| Search no. | Indexed and keywords terms |
| --- | --- |
| #1 | (pancreatic neoplasms OR pancreatic cancer OR pancreatic carcinoma OR pancreatic body and tail cancer OR left-sided pancreatic cancer OR pancreatic adenocarcinoma):ti,ab,kw |
| #2 | (radical antegrade modular pancreatosplenectomy OR RAMPS OR conventional distal pancreatectomy OR distal pancreatectomy OR distal pancreatosplenectomy OR CDP OR DPS):ti,ab,kw |
| #3 | #1 AND #2 |
| #4 | (randomized controlled trial OR RCT OR controlled clinical trial OR clinical trial OR comparative study OR cohort study OR retrospective study OR prospective study):ti,ab,kw |
| #5 | #3 AND #4 |
| #6 | NOT (review OR meta-analysis OR systematic review OR editorial OR letter OR case report):ti,ab,kw |
| #7 | #5 AND #6 |
| #8 | humans:ti,ab,kw |
| #9 | (adult OR middle aged OR aged):ti,ab,kw |
| #10 | #7 AND #8 AND #9 |

Database: CNKI

| Search no. | Indexed and keywords terms |
| --- | --- |
| #1 | SU = ('根治性顺行模块化胰脾切除术' + 'RAMPS' + '腹腔镜根治性顺行模块化胰脾切除术' + 'LRAMPS') AND SU = ('胰体尾癌' + '胰腺体尾部肿瘤' + '左侧胰腺癌' + '胰体尾恶性肿瘤') AND SU = ('传统胰体尾切除术' + '远端胰腺切除术' + '胰体尾联合脾切除' + 'DP' + 'DPS') |

Database: Wanfang

| Search no. | Indexed and keywords terms |
| --- | --- |
| #1 | (题名或关键词:"根治性顺行模块化胰脾切除术" OR "RAMPS") AND (题名或关键词:"远端胰腺切除术" OR "胰体尾切除术" OR "DP") AND (题名或关键词:"胰体尾癌" OR "左侧胰腺癌") |

Database: VIP

| Search no. | Indexed and keywords terms |
| --- | --- |
| #1 | (R=根治性顺行模块化胰脾切除术 OR R=RAMPS) AND (R=远端胰腺切除术 OR R=胰体尾切除术 OR R=传统手术) AND (R=胰体尾癌 OR R=左侧胰腺癌) |

Table S2 Risk of bias in included studies assessed using the ROBINS-I tool

| Author | Year | Type of bias | | | | | | | Overall rating |
| --- | --- | --- | --- | --- | --- | --- | --- | --- | --- |
|  |  | Confounding | Selection of participants | Exposure assessment | Misclassification during follow-up | Missing data | Measurement of the outcome | Selective reporting of the results |  |
| Latorre | 2013 | Moderate | Moderate | Low | Low | Moderate | Moderate | Low | Moderate |
| Lee | 2014 | Low | Moderate | Low | Low | Low | Low | Low | Moderate |
| Park | 2014 | Moderate | Moderate | Low | Low | Low | Low | Low | Moderate |
| Abe | 2016 | Moderate | Moderate | Low | Low | Low | Low | Low | Moderate |
| Kim | 2016 | Moderate | Moderate | Low | Low | Low | Low | Low | Moderate |
| Xu | 2016 | Moderate | Moderate | Low | Low | Low | Low | Low | Moderate |
| Wang | 2018 | Moderate | Moderate | Low | Low | Low | Moderate | Low | Moderate |
| Huo | 2019 | Moderate | Moderate | Low | Low | Low | Low | Low | Moderate |
| Yin | 2020 | Low | Moderate | Low | Low | Low | Low | Low | Moderate |
| Sham | 2020 | Moderate | Moderate | Low | Low | Moderate | Low | Low | Moderate |
| Dai | 2021 | Moderate | Moderate | Low | Low | Low | Low | Low | Moderate |
| Kim | 2021 | Low | Moderate | Low | Low | Low | Low | Low | Moderate |
| Niu | 2022 | Moderate | Moderate | Low | Low | Low | Low | Low | Moderate |
| Sutton | 2022 | Low | Moderate | Low | Low | Low | Low | Low | Moderate |
| Takahashi | 2023 | Low | Moderate | Low | Low | Low | Low | Low | Moderate |
| Zhu | 2023 | Moderate | Moderate | Low | Low | Low | Low | Low | Moderate |
| Borys | 2024 | Moderate | Moderate | Low | Low | Low | Moderate | Low | Moderate |
| Kwon | 2024 | Low | Moderate | Low | Low | Low | Low | Low | Moderate |
| Li | 2024 | Low | Moderate | Low | Low | Low | Low | Low | Moderate |
| Li-pen | 2024 | Moderate | Moderate | Low | Low | Low | Low | Low | Moderate |
| Yin | 2025 | Low | Moderate | Low | Low | Low | Low | Low | Moderate |

Supplementary Table S3. Certainty of the evidence

| **Outcome**  Timeframe | **Study results and measurements** | **Absolute effect estimates** | | **Certainty of the evidence**  (Quality of evidence) | **Summary** |
| --- | --- | --- | --- | --- | --- |
|  |  | CDP | RAMPS |  |  |
| Intraoperative transfusion rate | Relative risk: 0.79  (CI 95% 0.58 - 1.07)  Based on data from 1025 participants in 10 studies^1^ | **160**  per 1000 | **126**  per 1000 | **Low** | RAMPS may have little or no difference on intraoperative transfusion rate |
|  |  | Difference: **34 fewer per 1000**  (CI 95% 67 fewer - 11 more) | |  |  |
| R0 resection rate | Relative risk: 1.1  (CI 95% 1.03 - 1.18)  Based on data from 2304 participants in 19 studies^2^ | **737**  per 1000 | **811**  per 1000 | **Low** | RAMPS may improve R0 resection rate |
|  |  | Difference: **74 more per 1000**  (CI 95% 22 more - 133 more) | |  |  |
| Negative retroperitoneal margin rate | Relative risk: 1.2  (CI 95% 1.06 - 1.36)  Based on data from 999 participants in 8 studies^3^ | **661**  per 1000 | **793**  per 1000 | **Low** | RAMPS may improve negative retroperitoneal margin rate |
|  |  | Difference: **132 more per 1000**  (CI 95% 40 more - 238 more) | |  |  |
| Postoperative pancreatic fistula | Odds ratio: 1.01  (CI 95% 0.7 - 1.46)  Based on data from 1749 participants in 17 studies^4^ | **169**  per 1000 | **170**  per 1000 | **Low** | RAPMS may have little or no difference on postoperative pancreatic fistula |
|  |  | Difference: **1 more per 1000**  (CI 95% 44 fewer - 60 more) | |  |  |
| Postoperative bleeding | Odds ratio: 0.84  (CI 95% 0.5 - 1.41)  Based on data from 1747 participants in 15 studies^5^ | **36**  per 1000 | **30**  per 1000 | **Low** | RAMPS may have little or no difference on postoperative bleeding |
|  |  | Difference: **6 fewer per 1000**  (CI 95% 18 fewer - 14 more) | |  |  |
| Delayed gastric emptying | Odds ratio: 1.22  (CI 95% 0.74 - 2.03)  Based on data from 1327 participants in 12 studies^6^ | **42**  per 1000 | **50**  per 1000 | **Low** | RAMPS may have little or no difference on delayed gastric emptying |
|  |  | Difference: **9 more per 1000**  (CI 95% 11 fewer - 40 more) | |  |  |
| Chylous leakage | Odds ratio: 1.38  (CI 95% 0.75 - 2.53)  Based on data from 1160 participants in 9 studies^7^ | **37**  per 1000 | **50**  per 1000 | **Low** | RAMPS may have little or no difference on chylous leakage |
|  |  | Difference: **13 more per 1000**  (CI 95% 9 fewer - 52 more) | |  |  |
| Clavien-Dindo ≥ grade III complications | Odds ratio: 1.26  (CI 95% 0.8 - 1.97)  Based on data from 1267 participants in 14 studies^8^ | **57**  per 1000 | **70**  per 1000 | **Low** | RAMPS may have little or no difference on clavien-dindo ≥ grade iii complications |
|  |  | Difference: **14 more per 1000**  (CI 95% 11 fewer - 49 more) | |  |  |
| Total complications | Odds ratio: 0.83  (CI 95% 0.51 - 1.34)  Based on data from 1897 participants in 15 studies^9^ | **447**  per 1000 | **401**  per 1000 | **Low** | RAMPS may have little or no difference on total complications |
|  |  | Difference: **45 fewer per 1000**  (CI 95% 155 fewer - 73 more) | |  |  |
| Perioperative mortality | Odds ratio: 1.2  (CI 95% 0.31 - 4.66)  Based on data from 2564 participants in 20 studies^10^ | **3**  per 1000 | **3**  per 1000 | **Moderate** | RAMPS probably has little or no difference on perioperative mortality |
|  |  | Difference: **1 more per 1000**  (CI 95% 2 fewer - 11 more) | |  |  |
| OS | Hazard ratio: 0.93  (CI 95% 0.76 - 1.13)  Based on data from participants in 17 studies^11^ |  |  | **Low** | RAMPS may have little or no difference on os |
|  |  | Difference: **fewer** | |  |  |
| DFS | Hazard ratio: 1.01  (CI 95% 0.77 - 1.33)  Based on data from 0 participants in 14 studies^12^ |  |  | **Low** | RAMPS may have little or no difference on dfs |
|  |  | Difference: **fewer** | |  |  |
| Operation time | Measured by:  Scale: -  Based on data from 2145 participants in 20 studies^13^ |  |  | **Low** | RAMPS may have little or no difference on operation time |
|  |  | Difference: **MD 13.38 higher**  (CI 95% 12.58 lower - 39.33 higher) | |  |  |
| Intraoperative blood loss | Measured by:  Scale: -  Based on data from 2566 participants in 20 studies^14^ |  |  | **Low** | RAMPS may have little or no difference on intraoperative blood loss |
|  |  | Difference: **MD 43.37 lower**  (CI 95% 97.83 lower - 11.09 higher) | |  |  |
| Number of lymph nodes dissected | Measured by:  Scale: -  Based on data from 2417 participants in 18 studies^15^ |  |  | **Low** | RAMPS increases number of lymph nodes dissected |
|  |  | Difference: **MD 3.58 higher**  (CI 95% 1.66 higher - 5.5 higher) | |  |  |
| Length of hospital stay | Measured by:  Scale: -  Based on data from 1790 participants in 16 studies^16^ |  |  | **Low** | RAMPS may have little or no difference on length of hospital stay |
|  |  | Difference: **MD 0.60 lower**  (CI 95% 2.12 lower - 0.91 higher) | |  |  |

1. Systematic review [1] with included studies: Li-pen 2024, Zhu 2023, Kim 2016, Yin 2020, Abe 2016, Lee 2014, Latorre 2013, Dai 2021, Kwon 2024, Li 2024 **Baseline/comparator** Control arm of reference used for intervention .
2. Systematic review [1] with included studies: Yin 2025, Yin 2020, Kim 2021, Kwon 2024, Latorre 2013, Abe 2016, Li 2024, Takahashi 2023, Lee 2014, Park 2014, Dai 2021, Xu 2016, Zhu 2023, Sham 2020, Wang 2018, Huo 2019, Li-pen 2024, Kim 2016, Borys 2024 **Baseline/comparator** Control arm of reference used for intervention .
3. Systematic review [1] with included studies: Kim 2016, Yin 2025, Dai 2021, Lee 2014, Yin 2020, Li 2024, Li-pen 2024, Latorre 2013 **Baseline/comparator** Control arm of reference used for intervention .
4. Systematic review [1] with included studies: Lee 2014, Abe 2016, Wang 2018, Xu 2016, Niu 2022, Sham 2020, Dai 2021, Kwon 2024, Borys 2024, Kim 2016, Li 2024, Park 2014, Huo 2019, Takahashi 2023, Latorre 2013, Zhu 2023, Kim 2021 **Baseline/comparator** Control arm of reference used for intervention .
5. Systematic review [1] with included studies: Wang 2018, Lee 2014, Latorre 2013, Zhu 2023, Borys 2024, Park 2014, Xu 2016, Kim 2016, Li-pen 2024, Yin 2020, Abe 2016, Sham 2020, Dai 2021, Kwon 2024, Li 2024 **Baseline/comparator** Control arm of reference used for intervention .
6. Systematic review [1] with included studies: Lee 2014, Latorre 2013, Park 2014, Abe 2016, Kim 2016, Borys 2024, Kwon 2024, Dai 2021, Yin 2020, Zhu 2023, Yin 2025, Xu 2016 **Baseline/comparator** Control arm of reference used for intervention .
7. Systematic review [1] with included studies: Lee 2014, Sham 2020, Latorre 2013, Kim 2016, Xu 2016, Kwon 2024, Li 2024, Park 2014, Abe 2016 **Baseline/comparator** Control arm of reference used for intervention .
8. Systematic review [1] with included studies: Lee 2014, Kim 2021, Li 2024, Kim 2016, Zhu 2023, Takahashi 2023, Park 2014, Latorre 2013, Niu 2022, Li-pen 2024, Xu 2016, Borys 2024, Kwon 2024, Abe 2016 **Baseline/comparator** Control arm of reference used for intervention .
9. Systematic review [1] with included studies: Xu 2016, Wang 2018, Lee 2014, Sham 2020, Dai 2021, Kim 2016, Yin 2020, Park 2014, Li 2024, Li-pen 2024, Zhu 2023, Borys 2024, Yin 2025, Latorre 2013, Abe 2016 **Baseline/comparator** Control arm of reference used for intervention .
10. Systematic review [1] with included studies: Li-pen 2024, Zhu 2023, Takahashi 2023, Kim 2016, Park 2014, Borys 2024, Xu 2016, Dai 2021, Li 2024, Niu 2022, Abe 2016, Yin 2020, Lee 2014, Sham 2020, Kwon 2024, Latorre 2013, Yin 2025, Kim 2021, Wang 2018, Sutton 2022 **Baseline/comparator** Control arm of reference used for intervention .
11. Systematic review [1] . **Baseline/comparator** Control arm of reference used for intervention .
12. Systematic review [1] . **Baseline/comparator** Control arm of reference used for intervention .
13. Systematic review [1] with included studies: Wang 2018, Kim 2021, Huo 2019, Takahashi 2023, Sutton 2022, Li-pen 2024, Li 2024, Abe 2016, Latorre 2013, Lee 2014, Zhu 2023, Niu 2022, Dai 2021, Yin 2025, Yin 2020, Park 2014, Borys 2024, Xu 2016, Kim 2016, Kwon 2024 **Baseline/comparator** Control arm of reference used for intervention .
14. Systematic review [1] with included studies: Sham 2020, Lee 2014, Borys 2024, Kwon 2024, Yin 2020, Huo 2019, Dai 2021, Abe 2016, Xu 2016, Zhu 2023, Kim 2021, Niu 2022, Li 2024, Yin 2025, Sutton 2022, Kim 2016, Park 2014, Li-pen 2024, Wang 2018, Takahashi 2023 **Baseline/comparator** Control arm of reference used for intervention .
15. Systematic review [1] with included studies: Park 2014, Yin 2025, Yin 2020, Latorre 2013, Kim 2021, Wang 2018, Dai 2021, Li 2024, Kim 2016, Sham 2020, Zhu 2023, Huo 2019, Niu 2022, Sutton 2022, Kwon 2024, Takahashi 2023, Abe 2016, Li-pen 2024 **Baseline/comparator** Control arm of reference used for intervention .
16. Systematic review [1] with included studies: Lee 2014, Park 2014, Yin 2025, Kim 2021, Abe 2016, Sutton 2022, Kim 2016, Li-pen 2024, Latorre 2013, Dai 2021, Xu 2016, Huo 2019, Niu 2022, Zhu 2023, Li 2024, Kwon 2024 **Baseline/comparator** Control arm of reference used for intervention .

Supplementary Figure S1. Forest plot of comparison of RAMPS versus CDP for intraoperative blood loss.


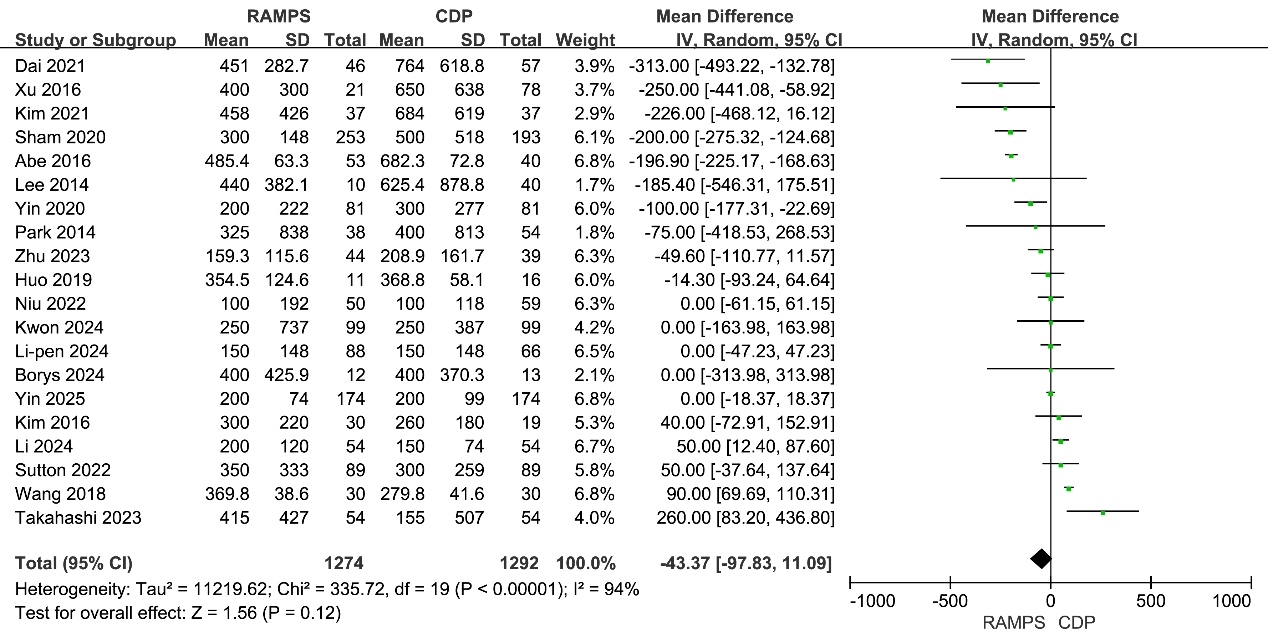


Supplementary Figure S2. Forest plot of comparison of RAMPS versus CDP for intraoperative transfusion rate.


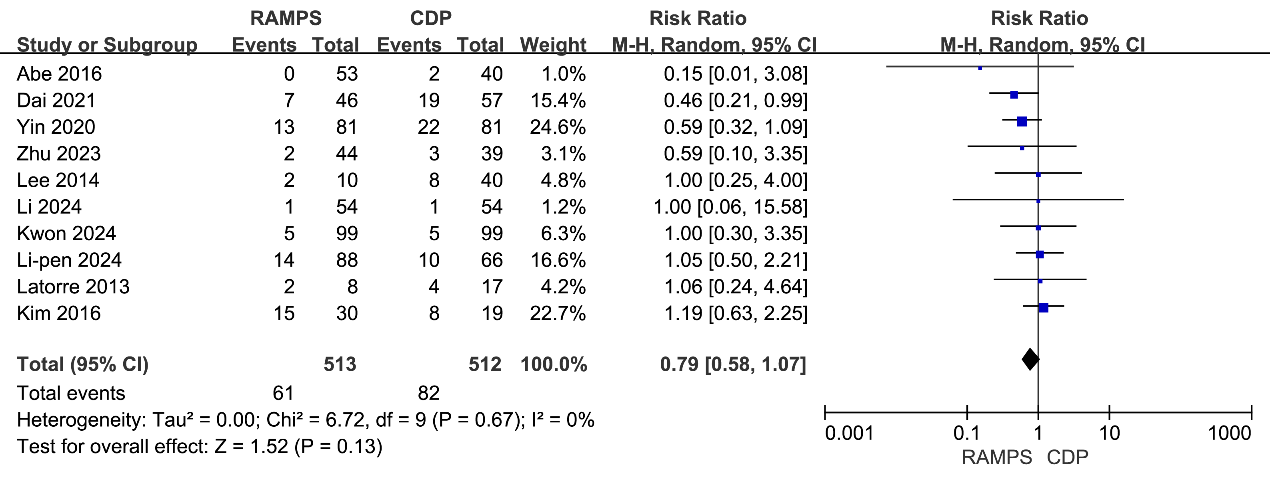


Supplementary Figure S3. Forest plot of comparison of RAMPS versus CDP for negative retroperitoneal margin rate.


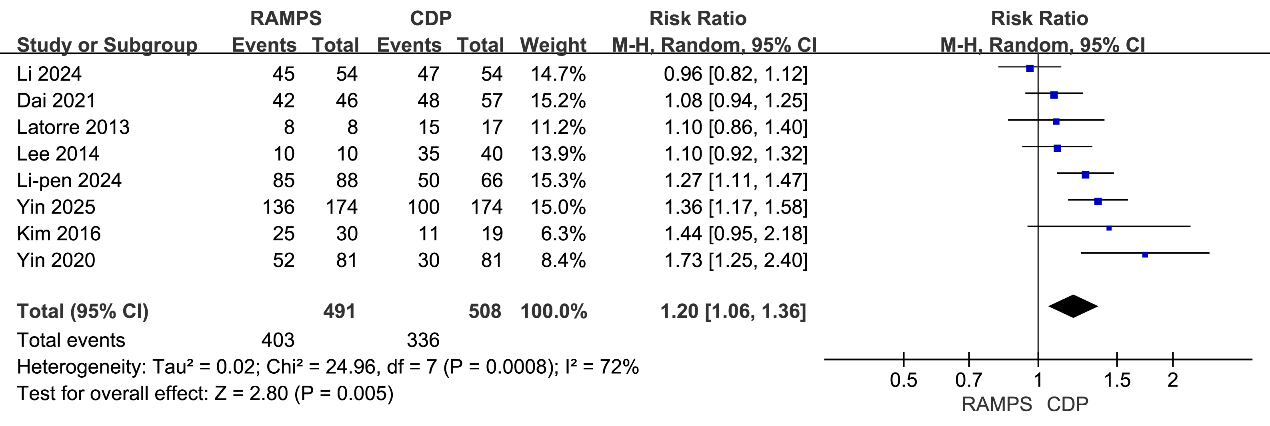


Supplementary Figure S4. Forest plot of comparison of RAMPS versus CDP for perioperative mortality.


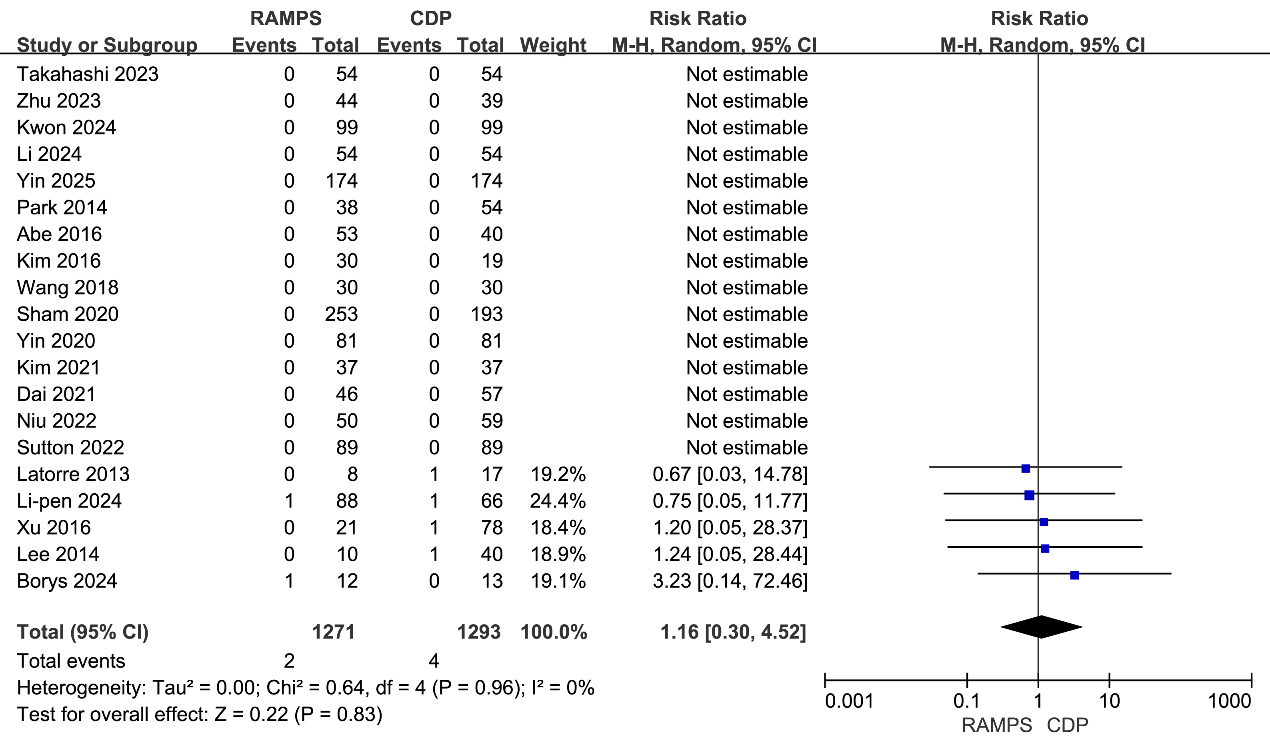


Supplementary Figure S5. Funnel plot and Egger test. (A) operative time; (B) intraoperative blood loss, (C) intraoperative transfusion rate; (D) length of hospital stay; (E) R0 resection rate; (F) number of lymph nodes dissected; (G) negative retroperitoneal margin rate; (H) overall survival; (I)disease-free survival; (J) perioperative mortality.


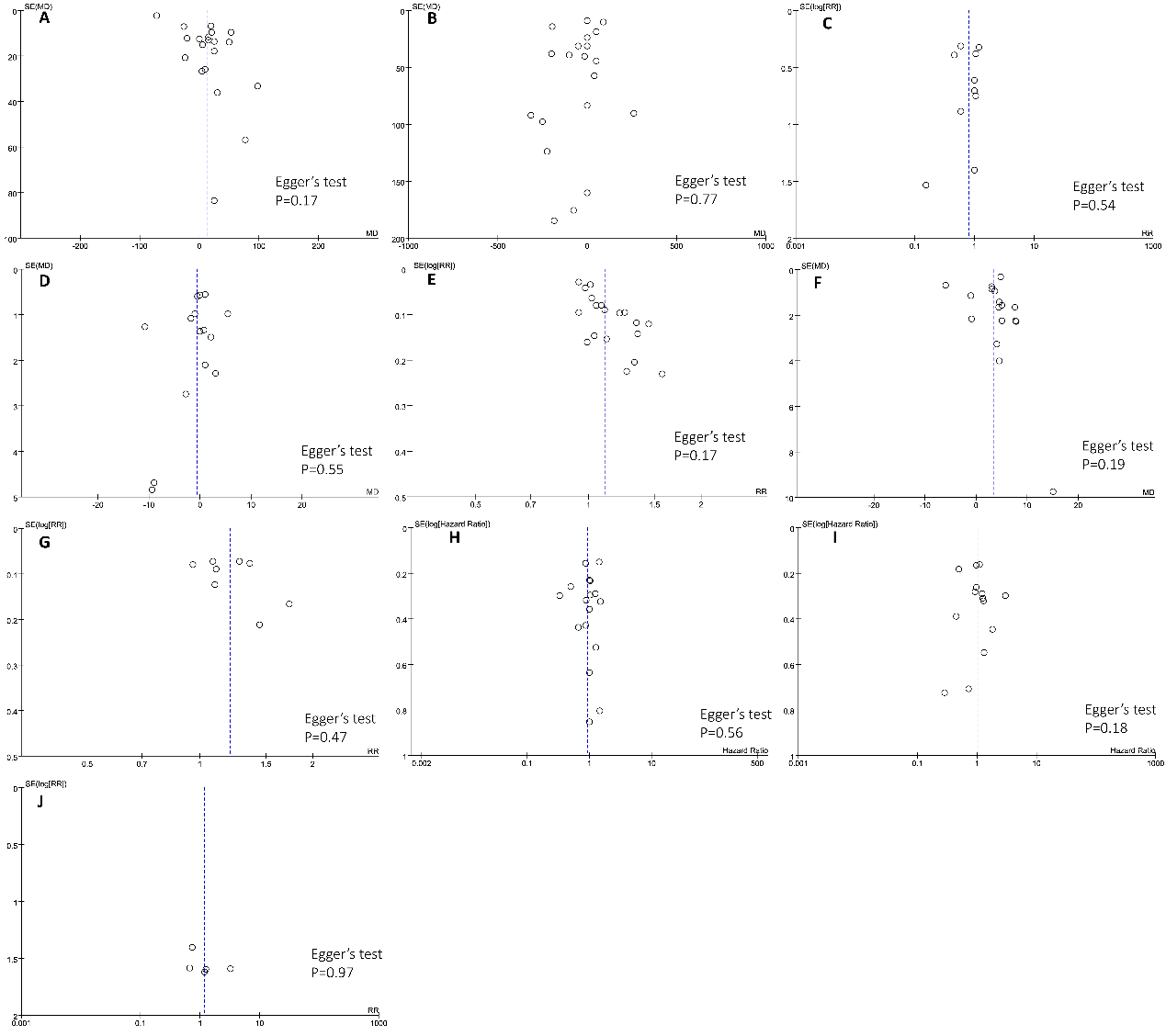

Supplement: Supplementary file 1 [file DataSheet1.docx]
